# Effects on Lignin Redistribution in *Eucalyptus globulus* Fibres Pre-Treated by Steam Explosion: A Microscale Study to Cellulose Accessibility

**DOI:** 10.3390/biom11040507

**Published:** 2021-03-29

**Authors:** Eduardo Troncoso-Ortega, Rosario del P. Castillo, Pablo Reyes-Contreras, Patricia Castaño-Rivera, Regis Teixeira Mendonça, Nicolás Schiappacasse, Carolina Parra

**Affiliations:** 1 Laboratorio de Recursos Renovables, Centro de Biotecnología, Barrio Universitario s/n, Universidad de Concepción, Concepción 4030000, Chile; rosariocastillo@udec.cl (R.d.P.C.); rteixeira@udec.cl (R.T.M.); roparra@udec.cl (C.P.); 2 ANID—Millennium Science Initiative Program-Millennium Nuclei on Catalytic Process towards Sustainable Chemistry (CSC), Av. Vicuña Mackenna 4860, Macul, Santiago 8320000, Chile; 3 Facultad de Ciencias Químicas, Barrio Universitario s/n, Universidad de Concepción, Concepción 4030000, Chile; 4 Facultad de Farmacia, Barrio Universitario s/n, Universidad de Concepción, Concepción 4030000, Chile; 5 Centro de Excelencia en Nanotecnología (CEN), Leitat Chile, Santiago 8320000, Chile; preyes@leitat.cl; 6 Unidad de Desarrollo Tecnológico UDT, Universidad de Concepción, Coronel 4190000, Chile; p.castano@udt.cl; 7 Facultad de Ciencias Forestales, Barrio Universitario s/n, Universidad de Concepción, Concepción 4030000, Chile; 8 Facultad de Ingeniería, Universidad Católica de Temuco, Temuco 4780000, Chile; lschiappacasse@uct.cl

**Keywords:** steam explosion, *Eucalyptus globulus*, lignin droplets, enzymatic hydrolysis, FT-IR imaging, chemometric analysis

## Abstract

The objective of this study was to investigate structural changes and lignin redistribution in *Eucalyptus globulus* pre-treated by steam explosion under different degrees of severity (S_0_), in order to evaluate their effect on cellulose accessibility by enzymatic hydrolysis. Approximately 87.7% to 98.5% of original glucans were retained in the pre-treated material. Glucose yields after the enzymatic hydrolysis of pre-treated material improved from 19.4% to 85.1% when S_0_ was increased from 8.53 to 10.42. One of the main reasons for the increase in glucose yield was the redistribution of lignin as micro-particles were deposited on the surface and interior of the fibre cell wall. This information was confirmed by laser scanning confocal fluorescence and FT-IR imaging; these microscopic techniques show changes in the physical and chemical characteristics of pre-treated fibres. In addition, the results allowed the construction of an explanatory model for microscale understanding of the enzymatic accessibility mechanism in the pre-treated lignocellulose.

## 1. Introduction

The recalcitrance of lignocellulosic biomass (LCB) to enzymatic hydrolysis is due to the interaction of the macromolecular components (cellulose, hemicelluloses, and lignin), which generates a rigid, fibrillar structure. For this reason, a pre-treatment process is necessary to break up the lignocellulosic matrix and increase its enzymatic digestibility in order to obtain monosaccharides and oligomers which will generate chemical building blocks or sugar to ferment to second-generation bioethanol [1,2,3]. Numerous methods have been developed for pre-treating LCB, including biological, physical, chemical, and physicochemical processes. Among the different pre-treatments available, steam explosion (SE) is commonly used for hardwoods, grasses, and agricultural residues [4,5,6,7]. SE has several attractive features when compared to other fractionation technologies: lower environmental impact, lower capital investment, higher energy efficiency, no use of hazardous chemicals, and controllable process conditions [2,8,9,10].

SE pre-treatment combines an autohydrolytic effect with mechanical disorganisation of the lignocellulosic fibre due to the effect of abrupt decompression of the LCB. It promotes breakdown of the lignocellulosic structure, hydrolysis of hemicelluloses, and depolymerisation of the lignin. In the first step, hydrolysis of the hemicelluloses is catalysed by the release of acetic acid from the acetylated xylans, initiated by the formation of hydronium ions generated from water [11,12]. The acid acts as a catalyst of hydrolytic reactions in wood polymers, promoting the solubilisation of hemicelluloses and partial degradation of lignin, and reducing the molar mass of the cellulose [13,14,15]. This process breaks down the long carbohydrate chains into oligomers and monomeric sugars. Lignin is primarily degraded through haemolytic cleavage of β-O-4 ether and other acid-labile linkages, producing a rearrangement of the molecule, and generating new stable carbon-carbon bonds [16,17]. These chemical modifications of the fibres have different impacts on cellulose accessibility because lignin and hemicelluloses are important limiting factors for enzyme accessibility and enzymatic hydrolysis. During pre-treatment, the hemicellulose is removed, and the lignin redistributed in large fibre extension; lignin and lignin fragments are rearranged and distributed heterogeneously in the cell wall, where they are deposited on the surface or interior of the fibres. Lignin micro-droplets are formed, produced by the coalescence and migration of lignin and progressive collapse of the microfibrils [18,19,20]. Other micro-particles deposited on the surface or interior of the fibre, called pseudo-lignin, consist of modified lignin and carbohydrate degradation products [21,22,23]. Both lignin micro-droplets and pseudo-lignin deposited on the surface or interior of the fibres reduce the efficiency of enzymatic hydrolysis through nonspecific binding of the enzymes, thus creating a physical barrier that blocks enzyme access to the cellulose [23,24].

However, it has also been proposed that the removal of hemicelluloses accompanied by the formation of lignin micro-droplets allows better enzyme action and catalytic adsorption to cellulose. As a result, there would be an increase in conversion rates during enzymatic hydrolysis because these droplets can migrate to certain regions of the cell wall, exposing the cellulose to the enzymes [19,25]. To demonstrate this proposal, in this study, *Eucalyptus globulus* woodchips were pre-treated by SE at different severity conditions. The main goal was to characterise the pre-treated material in order to evaluate, at microscale level, the enzyme accessibility of the pre-treated samples; and at the same time, to evaluate the possible role of the lignin micro-droplets in enzymatic saccharification. Microscopic techniques such as scanning electron microscopy (SEM), laser-scanning confocal fluorescence microscopy (LSCM), and FT-IR imaging were used. A possible action mechanism was proposed based on the results of the experiment.

## 2. Materials and Methods

### 2.1. Raw Material

Woodchips from 10–12-year-old *E. globulus* trees (donated by CMPC Cellulose S.A., Biobío, Chile) were screened to a size of 2.5 cm × 1.5 cm × 0.3 cm, air-dried to 10% moisture, and stored under dry conditions until use. The glucan content in the raw material was 45.5%, lignin 23.5%, xylans 15.3%, and acetyls 3.7% (see Table 1).

Additionally, we indicate for the raw material that there were 1.9% extractives in acetone and 2.0% ash. Ash content was determined by the TAPPI (Technical Association of Pulp and Paper Industry) method T 211 om-12.

### 2.2. Steam Explosion Process

SE was carried out in a 5 L SE reactor (SER) equipped with a 121 L expansion chamber. In each experiment, the SER was loaded with 200 g of *E. globulus* woodchips, dry wood basis (dwb). It was then fed with saturated steam, reaching 180, 200 or 220 °C (equivalent to 146, 230 or 348 psig, respectively), for a residence time of 2, 9.5, or 36 min (Table 1) before being depressurised to atmospheric pressure. The slurry was recovered and filtered, and the liquid was stored at 4 °C for further analysis of the glucose, xylose, and arabinose concentrations. To calculate the yield of solids (%), the pre-treated woodchips were air-dried for approximately 24 h, after which they were weighed, and the humidity content was determined in a fraction. These two data values were used to calculate the dry mass recovered after pre-treatment.

The severity factor ($S_{0}$), was calculated using the following equation [26]:

(1) $$S_{0}{\;=\;t\;*\;\exp[(T}_{H}-T_{R})/4.6]$$

where t is the reaction time in minutes, $T_{H}$ is the hydrolysis temperature in °C, and $T_{R}$ is a reference temperature of 100 °C. The value of 4.6 represents a constant, *ω*, which is an empirical parameter related with the activation energy and temperature of the reaction. Using a low value of *ω* (4.6) allows better correlation between the severity factor and the enzymatic digestibility of cellulose. In this case, the pre-treatment temperature has a greater influence than time reaction.

### 2.3. Chemical Characterisation of Samples

The woodchips were milled in a knife mill (Maestranza Proinco S.A., Concepción, Chile) and classified using a set of ASTM sieves (45–60 mesh). Milled wood was extracted with a 90% acetone solution for 16 h in Sohxlet apparatus to determine the amount of extractive. Steam-exploded samples were also milled and sieved, but not extracted with acetone. Milled samples were hydrolysed with 72% sulphuric acid at 30 °C for 1 h (300 mg of sample and 3 mL of sulphuric acid). The acid was diluted to 4% by the addition of 84 mL of water, and the mixture heated at 121 °C (1 atm.) for 1 h. The residual material was cooled and filtered through a number 4 porous glass filter. The solids were dried to constant weight at 105 °C and determined as insoluble lignin. The soluble lignin concentration in the filtrate was determined by measuring the absorbance at 205 nm, using the value of 110 L/g cm as the absorption coefficient. Glucose, xylose, and acetyl groups were determined by HPLC (Merck Hitachi) equipped with a refractive index detector and an Aminex HPX-87H (Bio-Rad, Hercules, CA, USA) column at 45 °C, eluted at 0.6 mL/min with 5 mM H_2_SO_4_ [27]. The factors used to convert sugar monomers to anhydromonomers were 0.90 for glucose (reported as glucan) and 0.88 for xylose (reported as xylans). These factors were calculated based on the water addition to polysaccharides during acid hydrolysis, as the molar mass of each original anhydromonomer in the polysaccharide. Thus, it increased 10% for glucose (from 162 g mol^−1^ in the anhydromonomer to 180 g mol^−1^ in glucose) and 12% for xylose (from 132 g mol^−1^ in the anhydromonomer to 150 g mol^−1^ in xylose). Acetyl content was calculated as the acetic acid content multiplied by 0.7. The percentages of reaction products (glucan, xylan, and lignin) in the solid and liquid fractions were calculated based on the theoretical initial amount of each component loaded into the SER (*w*/*w* dry basis). The moisture content of the solid fraction was determined using a moisture analyser (Sartorius MA35).

### 2.4. Scanning Electronic Microscopy (SEM)

Images were taken of the fibre surfaces before and after pre-treatment using a Jeol JSM-6380LV SEM instrument under a high vacuum, operating with a secondary electron detector. The samples were dried at room temperature and coated with conductive gold paint, particle size 500 Å, in a S150 Edwards Sputter Coater. Imaging was performed at a beam-accelerating voltage of 20 kV with a tungsten filament as the electron source.

### 2.5. Laser-Scanning Confocal Fluorescence Microscopy (LSCM)

A LSM710 confocal microscope (Axio Imager.Z1, Jena, Germany) was used with a ZEN 2008, using an excitation laser at Ar 488/° over an emission range of 490–560 nm and a 20× EC Plan Neofluar objective (N.A. 0.5) zoom 1.7. Software v. 5.0 (Zeiss); multichannel fluorescence images were acquired of the pulps obtained from pre-treatments at the different severities. The pulp samples were suspended in water and two drops of the suspension of each sample were placed on a slide for analysis. The samples were frozen with polyvinyl alcohol (Neg 50 Thermo Scientific) in order to obtain a cross section of the fibres at 10 µm on a cryostat Thermo Scientific model Microm HM 525 UV at −20 °C.

### 2.6. FT-IR Microimaging

FT-IR spectra and images of pulps were analysed by micro-FT-IR using an FT-IR Spectrum Frontier/Spotlight 400 Microscopy System (PerkinElmer, Inc. Waltham, MA, USA) with a linear array of mercury cadmium telluride (MCT) detectors. Areas between 45 µm × 70 µm and 70 µm × 60 µm of isolated fibres previously dried for 8 h at 45 °C were scanned by attenuated total reflectance (ATR), using a germanium crystal as the high reflective material, a pixel size of 1.56 µm × 1.56 µm, and a spectral range of 1000 to 1800 cm^−1^ with a spectral resolution of 8 cm^−1^ and 16 scans per spectrum. The spectra extracted from the images were subjected to ATR correction, removal of atmospheric noise, and baseline correction, and transformed to absorbance before imaging and chemometric analysis.

### 2.7. Chemometric Analysis

The images were subjected to multivariate analysis by principal component analysis (PCA) to detect the chemical components on the surface of the pre-treated materials. PCA was applied using Spectrum Image software (PerkinElmer Inc.), while the singular value decomposition (SVD) algorithm was performed in MATLAB (Math Works, Inc. Natick, MA, USA). Afterwards, a hypercube with two spatial coordinates related to *x*, *y* and a third spectral coordinate was unfolded in a matrix, and mean centring of the data was performed for pre-processing. The interactive method of self-modelling mixture analysis that is called “Pure” was employed for the ALS procedure. This is focused on selecting and using the purest variables as initial estimate values, and concentration as the direction of the variable selection. Non-negativity in spectra, concentrations, and in some cases unimodality, were used as constraints, with 50 iterations and a tolerance criterion for convergence of 0.1. The pure recovered spectra were then normalised by height.

Theory: A spectroscopic image can be displayed as a data cube with two dimensions related to the *x*, *y* coordinates of a scanned surface and a third spectral dimension. However, the measurement variation in an image dataset follows a bilinear model, where the mixed signal recorded in each pixel is described by the concentration-weighted sum of the pure signals of the chemical compounds. As a first step, an unfolding of the original image cube into a matrix of pixel spectra is required. Then, bilinear models can be performed followed by a refolding of the elements according to the original spatial structure of the image. MCR-ALS is a bilinear method of resolution, based on the mathematical decomposition of a global mixed instrumental response into the pure contributions due to each of the components in the system. One initial estimate, useful for spectroscopic images, is the estimation of purest variables (rows or columns) in the data matrix. This technique marking the most dissimilar row and column profiles in the raw measurements. The purest variables in the concentration direction provide the purest responses in the original measurements, and the purest response variables give the purest concentration profiles. An important consideration in using MCR algorithms is whether or not a unique solution (i.e., a solution with no ambiguity) can be obtained from a given dataset; therefore, some constraints are necessary. For example, the relative concentration and spectral intensity values cannot be negative; therefore, iterations between the MCR algorithms were carried out under non-negative constraints. The application of multivariate curve resolution with alternating least squares (MCR-ALS) for the analysis of FT-IR images is detailed in a previous publication [25].

### 2.8. Enzymatic Hydrolysis

Enzymatic hydrolysis was carried out in 250 mL Erlenmeyer flasks at 50 °C in a shaking incubator (Labtech LSI-4018A) at 150 rpm for 72 h. All experiments were run in triplicate with a total volume of 100 mL, composed of 10% solids (*w*/*v*), using a commercial cellulose enzyme complex (NS-22128 CCN3128; 71 FPU mL^−1^) supplemented with β-glucosidase (NS-22128 DCN00216; 265 CB mL^−1^) and 0.05 M sodium citrate pH 4.8 buffer. The enzyme dosages used were 20 FPU and 20 CBU of cellulase and β-glucosidase, respectively, per gram of dry material. The content of glucose released during enzymatic treatment was analysed by HPLC [28].

Enzymatic digestibility was measured using the following enzymatic hydrolysis yield:

$$EH\;yield\;=\;\frac{\left(\frac{Gs}{dry\;substrate\;loading\;\left(g\right)}\right)\;\times \;\%SR}{Gi}\;\times \;100$$

where Gs is the amount of glucose released (grams) from the dry substrate loading of pre-treated biomass, %SR is the percentage of solid recovered after the pre-treatment, and Gi is the initial amount of glucose in wood expressed as grams of monomer. All measurements were performed in triplicate.

## 3. Results and Discussion

### 3.1. Characterisation of Wood and Pre-Treated Materials

The chemical characterisations of *E. globulus* woodchips used for SE pre-treatment and pre-treated materials at different S_0_ conditions are shown in Table 1. The yield of solids recovered after SE ranged between 67.3% and 86.6%. The amount of xylans dissolved during the pre-treatment increased with the severity factor due to the solubilisation of hemicelluloses and lignin in accordance with the chemical characterisation of the liquid phase shown in Table 1. The xylan content in the liquid phase increased due to acid hydrolysis that occurs in hydrothermal pre-treatments of hardwoods, where the initial content of acetyls in the raw material acts as a catalyst. The lignin content in the liquid phase varied between 1.0% and 2.4%, not finding a clear trend between these variations and the severity factor. However, the range of lignin values in the liquid phase was low. The amount of residual xylans obtained in the materials subjected to pre-treatment between S_0_ 8.5 and 12.3 was 7.1% to 0.3%, approximately 46% and 1.9%, respectively, of the original amount in the wood. This result agrees with the appearance of the acetyl group in the liquid phase (Table 1) at the same severities. The solubility of xylans depends on the molecular weight and the presence of side chain substituents. Chen et al. (2010) mention that acetyl groups, arabinose, and uronic acid increase the solubility of xylans [29]. Other authors describe SE pre-treatment as an effective method for reducing LCB recalcitrance by removing hemicellulose, disrupting the lignin–hemicellulose matrix, and redistributing lignin in the cell wall layers [13,28], and thus allowing the preservation of glucans under controlled conditions. As the S_0_ of pre-treatment was increased from 8.5 to 12.3, glucan preservation varied between 98.2% and 90.3%, while lignin content in the same pre-treated materials varied between 22.5% and 25.9% (Table 1), slightly higher than the lignin content in the raw material. As mentioned above, the reason for this increase could be the generation of pseudo-lignin during SE pre-treatment. Araya et al. (2015) indicated that the increase in the lignin content of autohydrolysis pre-treated materials at higher severity conditions was partly due to the concomitant loss of polysaccharides and the formation of condensed lignin products. This increase could also be due to the formation of lignin-like compounds from lignin and carbohydrate degradation [21,22,23].

### 3.2. Enzymatic Hydrolysis

**Figure 1:**
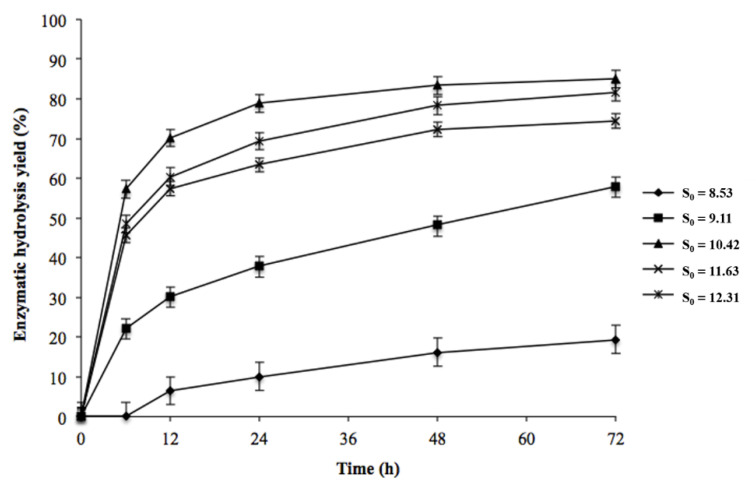
Kinetic profiles of enzymatic hydrolysis of pre-treated solids of *E. globulus* obtained by steam explosion pre-treatment at different severity factors. The glucans-to-glucose conversion yield is expressed on a dry wood basis. Each sample was performed in triplicate and the error bars correspond to the standard deviation of the repetitions.

The enzymatic digestibility and glucose yields of materials from *E. globulus* pre-treated with SE were evaluated, and the results are shown in Figure 1. In general, the efficiency of enzymatic digestibility increased with the S_0_; for instance, the enzymatic conversion of glucans to glucose increased from 19.4% to 85.1% when the S_0_ of the pre-treatment was increased from 8.5 to 10.4. The effect of SE pre-treatment on the glucose yield was attributable to the solubilisation of hemicellulose and the cleavage of lignin–carbohydrate bonds, disrupting the barriers which limit enzymes’ access to cellulose [24]. At a severity greater than 11.6, no positive effect was observed on the enzymatic hydrolysis yield; on the contrary, the saccharification yield decreased by more than 10%. This decrease can be attributed to the carbohydrate loss at higher severities. In order to analyse the effect of pre-treatment on the efficiency of enzymatic hydrolysis, we have plotted the glucose release kinetics in terms of the maximum potential glucose that comes from the original biomass, i.e., on a dry wood basis. When considering the yield of solids recovered after pre-treatment, we are considering the loss of glucans which are presented in Table 1, where it is indicated that the raw material contains 45.5% glucans, and as the severity of the pre-treatment increases, the glucans decrease until reaching 41.1% at the highest severity (S_0_ = 12.3). Most of the fermentable sugars present in the pre-treated materials were released in the first 48 h of enzymatic hydrolysis.

### 3.3. Microscopic Characterisation of Pre-Treated Material

**Figure 2:**
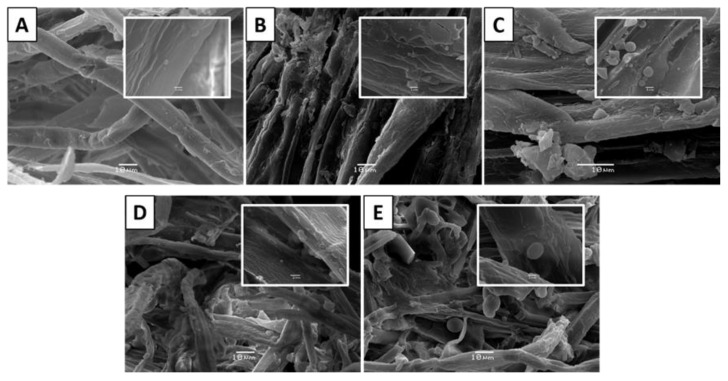
Surface images obtained by scanning electronic microscopy (SEM) of *E. globulus* pre-treated by steam explosion at different severity factors. (**A**) S_0_ = 8.53; (**B**) S_0_ = 9.11; (**C**) S_0_ = 10.42; (**D**) S_0_ = 11.63; (**E**) S_0_ = 12.31. The insert shows splits, delamination, cracks, and changes in the structure of the fibres and the appearance of lignin droplets. Bar size: 10 µm; bar size in the inserted micro-image: 1 µm.

To support the above statements on lignin modification and relocation within fibres in the form of droplets, samples were examined by scanning electron microscopy (SEM). The images revealed how the fibrous surface of the pre-treated material changed as the S_0_ of pre-treatments increased from 8.5 to 12.3 (Figure 2).

**Figure 3:**
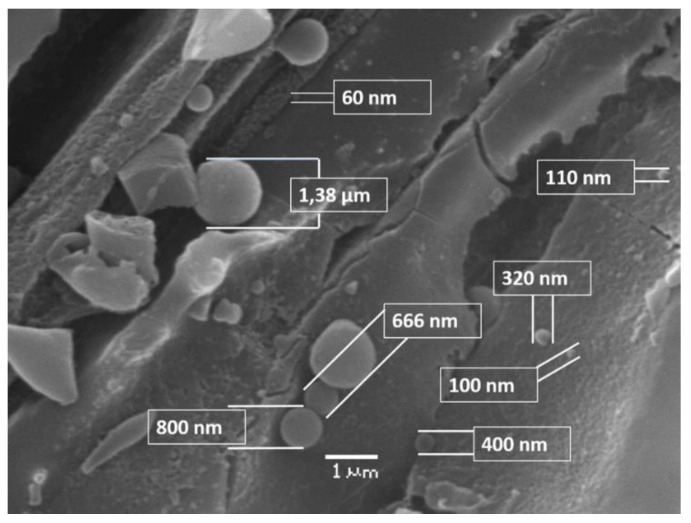
Diameter determination of lignin droplets formed during steam explosion pre-treatment at severity factor (S_0_) of 10.42 using SEM image. The diameters range between 60 nm and 1380 nm. Bar size: 1 µm.

The sudden depressurisation leads to an “explosion” of the steam inside the lignocellulosic matrix, which promotes the breakdown and defibrillation of its structure, hydrolysis of the hemicellulose, and depolymerisation/repolymerisation of lignin [28,30]. As the severity factor of SE pre-treatment increased, the SEM micrographs of the pre-treated material showed that the fibres were deconstructed, forming an amorphous cellulosic material similar to that observed by Gourlay et al., (2012) [31]. For example, fibre obtained at S_0_ 8.5 (Figure 2A) maintained its compact cellular structure, whereas woodchips treated at S_0_ 9.1 (Figure 2B) showed changes in the structure of the fibre with delimitation and mechanical damage. Figure 2C shows that SE produced noticeable physical changes in the structure of the fibres obtained at S_0_ 10.4, with the presence of micro-particles and micro-droplets between 60 nm and 1.38 µm in diameter (Figure 3). The formation of discrete droplets on the cell wall surface could be caused by the lignin flowing due to the high pre-treatment temperature, and later re-precipitating on the collapsed microfibrils, and by insoluble oligomers generated in acidic conditions that polymerise with degradation products from sugars and lignin. The distribution, size, and abundance of these droplets over the fibre varied, depending on the pre-treatment severity, which is consistent with the observations reported previously by other authors [19,20,32]. Woodchips subjected to the highest S_0_, at 11.6 and 12.3 (Figure 2D,E, respectively), suffered the greatest fibre damage, with changes in fibre length, width, or lumen diameter. By correlating the information obtained by SEM, the yields in enzymatic hydrolysis and chemical characterisation, we can show that the degradation of the fibre structure increases when the xylan content in the pre-treated solids decreases. On the other hand, we observed that there was a maximum performance of enzymatic hydrolysis at partial degradations of the fibres produced to severe intermediates.

**Figure 4:**
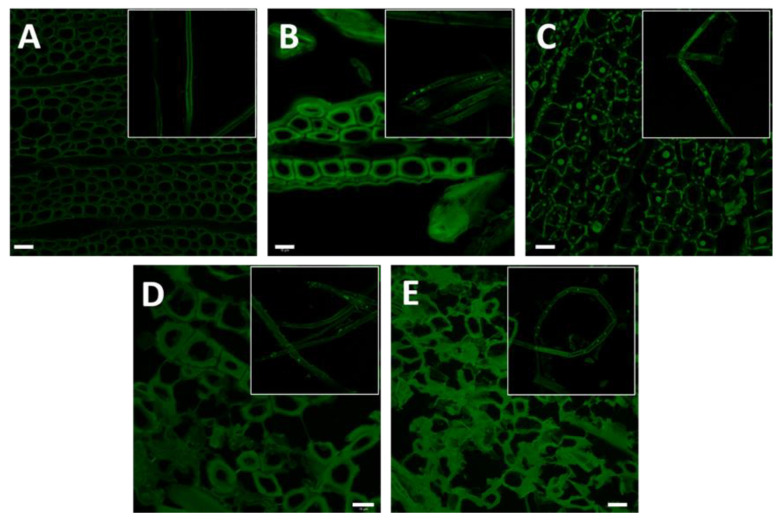
Laser-scanning confocal fluorescence microscopy (LSCM) image of pre-treated materials obtained by steam explosion at different severity factors. (**A**) S_0_ = 8.53; (**B**) S_0_ = 9.11; (**C**) S_0_ = 10.42; (**D**) S_0_ = 11.63; (**E**) S_0_ = 12.31. Bar size: 10 µm. In the insert, the distribution of lignin along the fibre can be observed.

**Figure 5:**
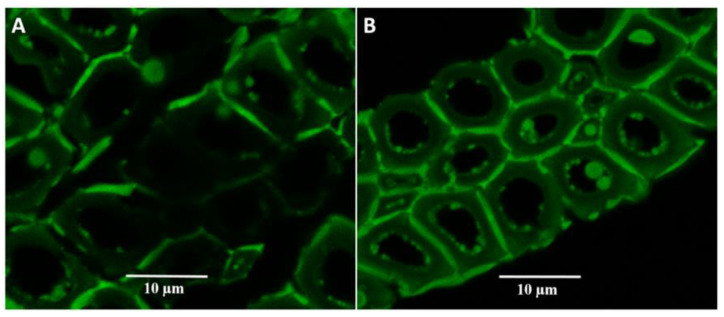
LSCM image. Images of areas with different lignin distributions in pre-treated material at severity S_0_ 10.42. (**A**) A zone with highly delignified fibres; and (**B**) micro and nano drops of lignin lodged inside the cell lumen.

The intrinsic autofluorescence of lignin (emission at 530 nm) can be used to assess its location on the cell wall using LSCM [19]. The distribution of fluorescence produced by the presence of lignin in materials subjected to different pre-treatments was observed in cross-sections and along the fibres, distributed mainly in the fibre wall. In the cross-section of material pre-treated at S_0_ 8.5, the lignin was observed to be distributed homogeneously in the cell wall and along the fibre, forming a physical barrier that would hinder enzyme access to the cellulose (Figure 4A). When the S_0_ of the pre-treatment was increased from 8.5 to 9.1, slight delignification of the cell wall (cross-section) was observed, as well as small spheres associated with the condensation of lignin along the fibre (Figure 4B). Figure 4C shows a cross section of material pre-treated at S_0_ 10.4; spheres of lignin were deposited in the lumen, which is an important porous structure for the diffusion and subsequent adsorption of enzymes. The close-up image (Figure 5A) shows that some areas of the cell wall have been delignified while others maintain a homogeneous lignin distribution. In Figure 5B, micro and nano drops of lignin can be clearly identified, lodged within the lumen of the cell; this generates areas of the cell wall with lower lignin content (low fluorescence) in which cellulose could be exposed for enzymatic hydrolysis. When the S_0_ of the pre-treatment was increased to 11.63 (Figure 4D) and 12.31 (Figure 4E), the lignin distribution was extensively modified. It is observed mainly in the cell wall, probably due to the re-condensation of lignin and carbohydrate degradation products. In the cross-section (Figure 4D), physical changes can be observed in the structure of the fibres, while some lignin spheres could be detected in the longitudinal image of the fibre. Figure 4E shows the presence of cracks in the fibre, exposing the internal cell walls, and the loss of cohesion within the fibres is mainly due to the high severity of the pre-treatment.

By LSCM analysis, it was possible to verify a significant redistribution of lignin throughout the cellular structure of the biomass. This is related to the increase in the content of xylans dissolved in the liquid phases obtained from the pre-treatment (Table 1), which would indicate that the action of the pre-treatment, favoured by the presence of acetyl in the medium, helps to dissociate the carbohydrate–lignin complex and, consequently, increases the solubilisation of xylans and the degree of distribution of lignin. It was expected that the enzymatic hydrolysis would improve when the solubilisation of xylans, the heterogeneous redistribution of lignin, and the degradation of the fibre structure increased. However, this is not what happens. It could be that another factor exists, which limits the complete hydrolysis, and it is only possible to reach a maximum glucose and at intermediate severities.

Fourier-transform infrared microspectroscopy was used to obtain chemical information about the surface of the pre-treated fibres, to evaluate the distribution patterns, and generate pure spectra of the lignocellulosic components in the fibres. This technique was supported by multivariate methods including principal component analysis (PCA) and multivariate curve resolution with alternating least squares (MCR-ALS). Samples from three of the five pre-treatments studied were selected, obtained at S_0_ conditions 8.5, 10.4, and 12.3. Three main components were used for the reconstructions of each sample.

**Figure 6:**
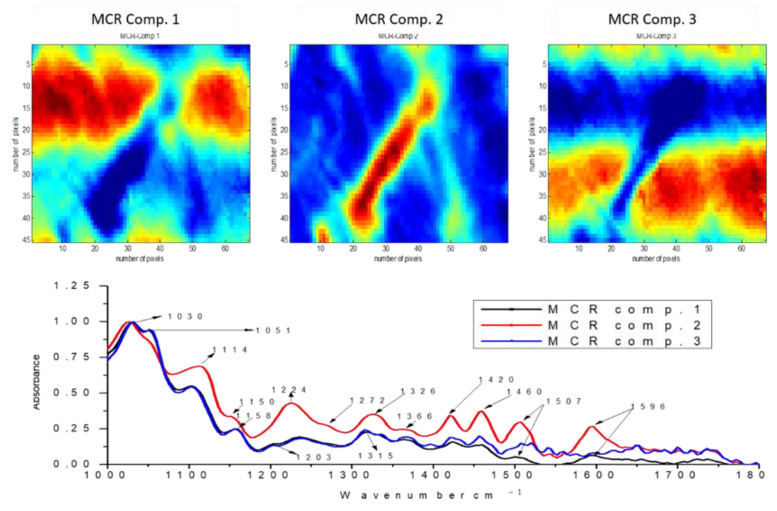
Multivariate curve resolution with alternating least squares (MCR−ALS) relative concentration and recovered spectra of chemical components of pre−treated materials obtained by steam explosion. FT−IR micro-image of *E. globulus* pre-treated at severity S_0_ 8.53.

The FT-IR spectra corresponding to three main components in the S_0_ 8.5 sample (Figure 6) show an intense band at 1030 cm^−1^, characteristic of cellulose [33]. The second component is characterised by intense bands at 1420 cm^−1^, 1507 cm^−1^ and 1596 cm^−1^, attributable to the aromatic ring in lignin [34]. These bands are also observed with less intensity in components 1 and 3, which indicates that the material has a mixture of lignocellulosic components on its surface, i.e., less chemical deconstruction of the cell wall.

**Figure 7:**
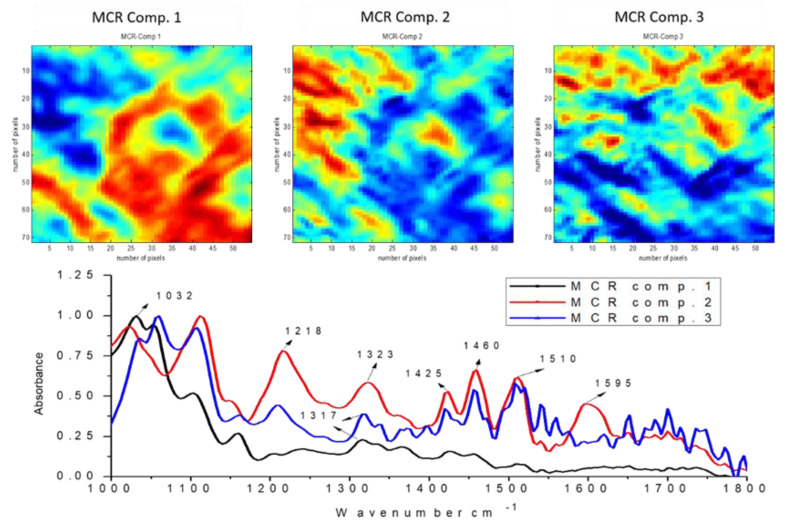
MCR−ALS relative concentration and recovered spectra of chemical components of pre−treated materials obtained by steam explosion. FT−IR micro-image of *E. globulus* pre-treated at severity S_0_ 10.42.

In the pre-treated material obtained at S_0_ 10.4 (Figure 7), an increase is observed in the relative concentration of lignin, shown by the increase in the intensity of the 1510 cm^−1^ and 1595 cm^−1^ bands. Components 1 and 3 continue to show an intense band at 1032 cm^−1^. An increase was also observed in the intensity of the 1327 cm^−1^ band, especially in the third component, which could mean greater separation of the component on the surface. In addition, in the spectrum of the second component, where the highest intensities of lignin-related bands are detected, an increase was found in the 1323 cm^−1^ band, indicating that it corresponds to a condensed G-type lignin, substituted in position 5 [35].

**Figure 8:**
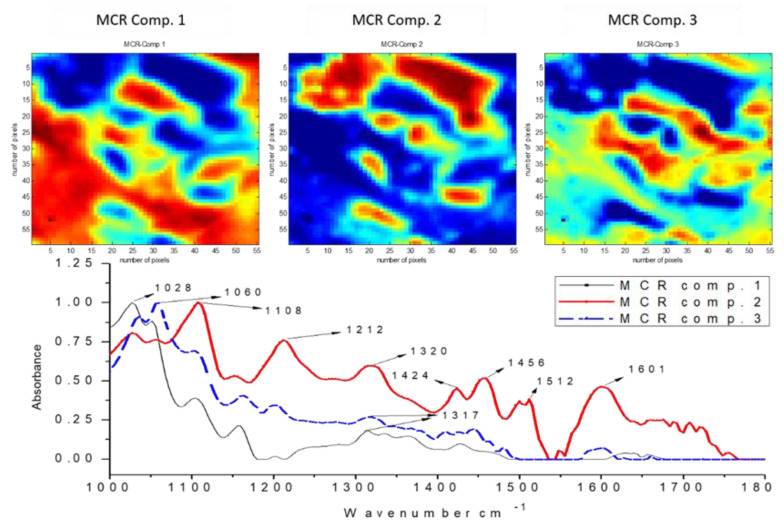
MCR−ALS relative concentration and recovered spectra of chemical components of pre−treated materials obtained by steam explosion. FT−IR micro-image of *E. globulus* pre-treated at severity S_0_ 12.31.

In the material pre-treated at the highest severity, significant displacement of the 1505 cm^−1^ band (observed at 1512 cm^−1^) and the 1595 cm^−1^ band (observed at 1601 cm^−1^) was identified in the spectrum of the second component (Figure 8). This indicates high chemical modification of lignin, due to the disruption of the propyl group and β-O-4 structures by the high severity of pre-treatment [36]. This causes lignin to be affected by condensation reactions to form more stable bonds such as 5–5, β–β or β–5, which are of the C–C type. In components 1 and 3, an intense band of cellulose is observed at 1028 cm^−1^, but in the spectrum of the second component, the intensity of this band decreases significantly, indicating greater deconstruction resulting from the increased severity. This can also be confirmed by the absence of aromatic lignin bands at 1512 cm^−1^ and 1601 cm^−1^ in the spectra of components 1 and 3.

The FT-IR image analysis provided information on the chemical modifications of the biomass components that occurred during pre-treatment. These results could help to respond to the lower enzymatic hydrolysis yields achieved by the materials of higher severity. The greater the severity, the greater the dissociation of biomass constituents that was observed, added to a greater degree of condensation of the redistributed lignin. This lignin can act as an inhibitor of cellulases by unproductive adsorption, reducing the catalytic activity of enzymes. This contributes to understanding why the best enzymatic hydrolysis yields are achieved at intermediate severities.

### 3.4. Study of Micro-Accessibility of E. globulus Pre-Treated by Steam Explosion

**Figure 9:**
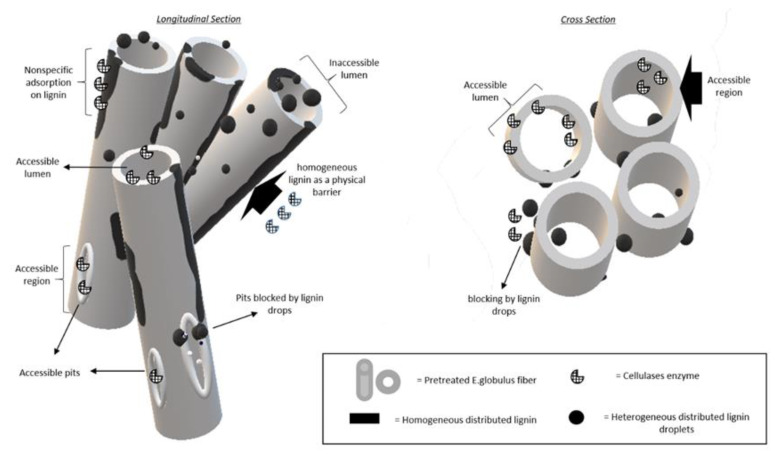
Hypothetical diagrammatic presentation of the effect of pre-treatment on the formation of micro and nano lignin droplets generating a heterogeneous distribution of lignin, increasing the areas of enzymatic microaccessibility and subsequent digestion of cellulose. Pre-treated fibres are represented lengthwise on the left and in cross section on the right. S_0_ 8.53.

The enzymatic hydrolysis yield is influenced by physicochemical and structural changes in the pre-treated material. A significant removal of hemicellulose in the biomass was determined, promoting both the degradation of the fibre structures and the redistribution of lignin. We can observe by SEM analysis that as the severity of the steam explosion increases, the degree of degradation of the fibres increases, probably related to the decrease in xylans in the pre-treated materials. By LSCM analysis, it was possible to verify a significant redistribution of lignin throughout the cellular structure of the biomass. This is related to the increase in the content of xylans dissolved in the liquid phases obtained from the pre-treatment (Table 1), which would indicate that the action of the pre-treatment, favoured by the presence of acetyl in the medium, helps to dissociate the carbohydrate–lignin complex, and, consequently, increasing the solubilisation of xylans and increasing the degree of distribution of lignin. To represent this phenomenon, Figure 9 shows a hypothetical model of the micro-accessibility mechanism proposed to explain the changes inside the fibres, where the effect of SE pre-treatment on the heterogeneous distribution of lignin after pre-treatment is shown. When hemicelluloses are removed, lignin is more exposed to changes that promote its redistribution in the form of micro-droplets; these are randomly relocated in different regions of the fibre surface or inside the cell wall, leaving the cellulose more exposed to enzymes. At low temperature, the lignin is insoluble in water due to the high dielectric constant of water (78.5 at 25 °C); however, at the extreme conditions of temperature and pressure used in the pre-treatments, the dielectric constant of water decreases dramatically [37], generating a favourable environmental for the solubilisation of lignin. Subsequently, during the cooling process, the dielectric constant of water is restored, causing the precipitation and formation of lignin micro-droplets on the fibres [32].

## 4. Conclusions

This work demonstrates that the severity conditions employed during the SE pre-treatment of *E. globulus* influenced the enzymatic hydrolysis of the pre-treated material cellulose. The microscopy analysis showed that a relationship exists between the SE pre-treatment severity conditions and the degradation degree of the fibre structures, the heterogeneous redistribution of lignin, and the chemical changes of redistributed lignin. With respect to lignin redistribution, it was observed on the surface and the interior of the *E. globulus* fibre, in the form of micro and nanodroplets, and hence the cellulose of the fibre was more exposed. Moreover, with respect to the chemical changes of redistributed lignin, it presented a higher degree of condensation as severity increased.

These physical–chemical effects were correlated with a greater solubilisation of xylans from biomass to the liquid phase, due to the action of acetyl groups of the raw material. The solubilisation of the xylans could be the main phenomenon that leads to the dissociation of the carbohydrate–lignin complex, promoting the degradation of the fibre structure and the redistribution of lignin. The migration and redistribution of lignin in the residual biomass significantly improved enzymatic hydrolysis.

On the other hand, it was determined that the maximum conversion to glucose was achieved at conditions of intermediate severity. At higher severity conditions, the condensation of lignin increased, which caused an unproductive adsorption of the enzymes on it. Based on the experimental evidence found, a model was proposed that explains the effect of pre-treatment with SE in lignocellulosic material fibre and its impact on enzymatic hydrolysis.

## Figures and Tables

**Table 1:** Chemical composition of wood, pulps and liquid fractions obtained from steam explosion pre-treatment at different severities.

		Pre-Treated Materials				
Sample	Raw Material	1	2	3	4	5
Temperature, °C	--	180	180	200	220	220
Time, min	--	9.5	36	9.5	2.0	9.5
S_0_, ω = 4.6	--	8.5	9.1	10.4	11.6	12.3
Solids recovered, %		86.6	77.1	72.3	69.4	67.3
Glucans, %	45.5	44.3 ± 0.8	44.5 ± 0.3	43.4 ± 0.8	42.1 ± 1.0	41.1 ± 0.5
Xylans, %	15.3	7.1 ± 0.9	5.9 ± 0.3	3.4 ± 0.4	2.0 ± 0.1	0.3 ± 0.1
Lignin, %	23.5	23.9 ± 0.4	23.0 ± 0.1	22.5 ± 0.8	25.1 ± 0.4	25.9 ± 0.1
Acetyl groups, %	3.6	nd	nd	nd	nd	nd
		**Liquid phase**				
Glucans, %	--	0.1± 0.0	0.3 ± 0.0	0.4 ± 0.0	0.1 ± 0.0	1.1 ± 0.0
Xylans, %	--	3.5 ± 0.1	8.5 ± 0.1	11.2 ± 0.2	13.8 ± 0.2	15.6 ± 0.1
Arabinans, %	--	0.1 ± 0.0	0.1 ± 0.0	0.3 ± 0.0	0.2 ± 0.0	0.1 ± 0.0
Acetyl groups, %		0.6 ± 0.0	2.0 ± 0.1	3.0 ± 0.0	3.4 ± 0.1	3.2 ± 0.1
Lignin, %	--	1.0 ± 0.1	2.4 ± 0.3	2.3 ± 0.3	1.5 ± 0.2	2.1 ± 0.1
Formic acid	--	nd	0.2 ± 0.0	0.5 ± 0.0	0.2 ± 0.0	0.3 ± 0.0
HMF	--	nd	0.1 ± 0.0	0.2 ± 0.0	0.2 ± 0.0	0.2 ± 0.0
Furfural	--	0.1 ± 0.0	0.6 ± 0.0	1.7 ± 0.1	1.1 ± 0.1	0.7 ± 0.0

S_0_: severity factor; ω: empirical parameter related to activation energy and temperature; HMF: 5-hydroxymethylfurfural.; nd: not detected; all concentrations are on a dry wood basis.

## Data Availability

Not applicable.
